# Statin use, hyperlipidaemia, and the risk of breast cancer

**DOI:** 10.1038/sj.bjc.6600267

**Published:** 2002-05-06

**Authors:** J A Kaye, C R Meier, A M Walker, H Jick

**Affiliations:** Department of Epidemiology, Harvard School of Public Health, 677 Huntington Avenue, Boston, Massachusetts, MA 02115, USA; Boston Collaborative Drug Surveillance Program, Boston University School of Medicine, 11 Muzzey Street, Lexington, Massachusetts, MA 02421, USA; Basel Pharmacoepidemiology Unit, Division of Clinical Pharmacology and Toxicology, University Hospital, University of Basel, Petersgraben 4, CH-4031 Basel, Switzerland

**Keywords:** breast cancer, HMG Co-A reductase inhibitors, statins, hyperlipidaemia, case–control study

## Abstract

Hydroxymethyl glutaryl coenzyme A inhibitors (‘statins’) are carcinogenic in rodents and an increased incidence of breast cancer was reported among pravastatin users in one randomised trial. We conducted a case–control study in the General Practice Research Database to evaluate the risk of breast cancer among 50- to 79-year old women treated with statins for hyperlipidaemia. Case and control women were matched by age, general practice, duration of prescription history in the General Practice Research Database, and index date. Adjusting for history of benign breast disease, body mass index, and use of hormone replacement therapy, women currently treated with statins had an estimated relative risk for breast cancer of 1.0 (95% confidence interval 0.6–1.6) compared to women without hyperlipidaemia. Untreated hyperlipidaemia was associated with an increased risk of breast cancer (estimated relative risk 1.6; 95% confidence interval 1.1–2.5). The estimated relative risk among women currently receiving only non-statin lipid-lowering drugs was similar to that of women with untreated hyperlipidaemia (1.8; 95% confidence interval 0.9–3.4). We found no evidence for an increasing trend in breast cancer risk with increasing duration of statin use (median duration 1.8 years, maximum 8.6 years).

*British Journal of Cancer* (2002) **86**, 1436–1439. DOI: 10.1038/sj/bjc/6600267 www.bjcancer.com

© 2002 Cancer Research UK

## 

Hydroxymethyl glutaryl coenzyme A reductase inhibitors (‘statins’) and fibrates (the other leading class of cholesterol-lowering drugs) can cause cancer in rodents (Newman and Hulley, 1996). Clinical trials focused on cardiovascular mortality and morbidity have had limited ability to assess the human carcinogenic potential of statins due to relatively short follow-up. Among 13 trials reporting cancer incidence and deaths as outcomes, with an average follow-up time of 3.3 years, no evidence was found of an overall increased risk of cancer (relative risk (RR) 1.03, 95% confidence interval (CI) 0.90–1.17) or cancer deaths (RR 0.95, 95% CI 0.74–1.21) among statin users (Hebert *et al*, 1997). However, concern was raised by the only study that reported on breast cancer risk, with 12 cases among pravastatin users and only one case in a placebo group of similar size (*P*=0.002) (Sacks *et al*, 1996).

We used the General Practice Research Database (GPRD), an automated data source containing drug prescription and other medical information on more than 3 million residents of the UK, to study the possible relation of breast cancer to statin use and hyperlipidaemia.

## MATERIALS AND METHODS

We conducted a matched case–control study on the risk of breast cancer in relation to use of statins, non-statin lipid-lowering agents, and untreated hyperlipidaemia among women aged 50–79 diagnosed with breast cancer in 1992–1998 in the UK General Practice Research Database (GPRD).

### General Practice Research Database (GPRD)

The GPRD started in 1988 and has been used extensively for research studies; the subset of data we have used during the past decade is of consistently high quality. (Jick *et al*, 1991) Some 350 practices contributed to data used in this report. In a previous study our group found that diagnoses of cancer are validated in 95% of cases for whom further information is obtained from general practitioners (Jick *et al*, 1997). Thirty of 30 randomly selected probable cases of breast cancer were confirmed in an earlier review of their pathology report or hospital discharge summary (Meier *et al*, 2000). Also, we recently reported that the age-adjusted incidence rates of breast cancer among women in the GPRD are closely similar to those reported by the UK Office of National Statistics (Kaye *et al*, 2000).

### Base population

The base population consisted of subjects born from 1920 to 1955 for whom at least one lipid-lowering drug prescription (see Appendix) was recorded in the GPRD (*n*=52 924); subjects born in the same years who had a recorded diagnosis of hyperlipidaemia (ICD-8 codes 272.0, 272.1, and 272.3) but no recorded prescription for any lipid-lowering drug (*n*=18 189); and a random sample of 50 000 women born in the same years who had no recorded diagnosis of hyperlipidaemia and no recorded prescription for any lipid-lowering drug. We excluded women who underwent bone marrow or other organ transplantation, received cyclosporine, or had HIV infection or AIDS.

### Cases and controls

From the base population we identified all women with newly diagnosed breast cancer at age 50–79. Because *in situ* and invasive cancers are not coded separately in the database, both were included in this study. On the basis of individual review of computer profiles in which prescription information was suppressed, we excluded cases with a prior history of breast or other cancer, those in whom the diagnosis of breast cancer appeared uncertain, and those first diagnosed with breast cancer at the time of death. We also excluded cases with less than 5 years of recorded prescription information in the GPRD.

From the base population we matched up to five controls to each case by sex, year of birth (within 2 years), date of first recorded prescription in the GPRD (within 1 year), and general practice. Controls were required to have evidence of active follow-up in the database on the date their matched case was diagnosed (‘index date’). The same exclusions were applied to the controls as to the cases.

### Exposure assessment

The use of a lipid-lowering agent was ‘current’ if a subject had at least one prescription within 6 months before the index date. Past use was defined as having at least one recorded prescription more than 6 months before the index date. Subjects who were current or past users of both statins and non-statins were classed as current or past statin users only. Untreated hyperlipidaemia was defined as a recorded diagnosis of hyperlipidaemia (see ‘Base population’ for diagnostic codes) at any time before the index date in the absence of treatment.

To assess the effect of duration of treatment for current users, we calculated the time interval from each subject's first prescription for a given class of drug to her last prescription plus the number of days covered by the last prescription up to the index date.

### Other covariates

We evaluated body mass index (BMI), current or past hormone replacement therapy (HRT), and history of benign breast disease as potential confounding factors. BMI was calculated from the first recorded height and weight measurement in each subject's computer record, and the measurement of weight must have preceded the index date. We considered a woman to have a history of benign breast disease if she had a diagnosis of fibrocystic disease, intraductal papilloma, or fibroadenoma recorded at any time before her index date. We defined HRT as any recorded prescriptions for systemic oestrogen (including patches but excluding creams) either alone or in combination with a progestin. We distinguished current and past users according to whether they received any HRT prescriptions within 6 months before their index date.

Subjects were designated by coded GPRD record numbers and we had no access to personal identifying information. The study protocol was approved by the Scientific and Ethical Advisory Group of the GPRD.

### Statistical methods

We used conditional logistic regression to estimate odds ratios and 95% confidence intervals. The reference group for model-adjusted analyses comprised women who had no diagnosis of hyperlipidaemia, had no recorded prescriptions for a lipid-lowering agent before their index date, did not use hormone replacement therapy, had low body mass index (<24 kg m^2^) and had no history of benign breast disease. We report estimated odds ratios as ‘relative risks’ with 95% confidence limits. Statistical calculations were performed using SAS, version 8.01 (SAS Institute, Inc., Cary, NC, USA).

## RESULTS

We identified 224 women aged 50 to 79 with incident breast cancer in the base population and 1009 controls matched by age, sex, general practice, duration of prescription drug history in the GPRD, and index date.

Characteristics of the cases and controls are listed in Table 1

**Table 1:**
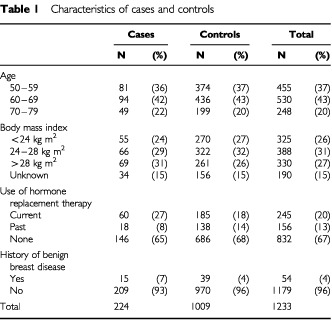
Characteristics of cases and controls

. Body mass index (BMI) was greater than 28 kg m^2^ in a higher proportion of cases than controls. Although hormone replacement therapy (HRT) was used by approximately the same proportion of cases as controls, current HRT use was more frequent among cases. Cases were more likely than controls to have a history of benign breast disease.

Table 2

**Table 2:**
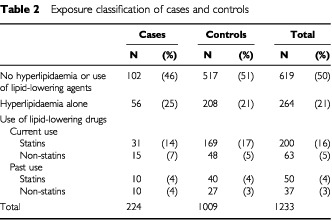
Exposure classification of cases and controls

lists the proportions of cases and controls with a recorded history of hyperlipidaemia and treatment with lipid-lowering drugs. Untreated hyperlipidaemia was more frequent among cases than controls.

Multivariable model-adjusted relative risks for breast cancer according to exposure group are presented in Table 3

**Table 3:**
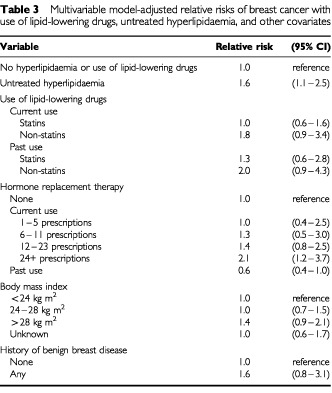
Multivariable model-adjusted relative risks of breast cancer with use of lipid-lowering drugs, untreated hyperlipidaemia, and other covariates

. Compared with women without hyperlipidaemia, the relative risk (RR) for breast cancer among women currently treated with statins was 1.0 (0.6–1.6). Among the 200 current statin users, 26 were current users of pravastatin (two cases and 24 controls), the RR among women currently treated with pravastatin compared to women without hyperlipidaemia was 0.4 (0.1–1.8).

Compared to women without hyperlipidaemia, the risk was modestly increased among women with untreated hyperlipidaemia (RR 1.6 (1.1–2.5)) and among those with hyperlipidaemia currently treated with non-statin lipid-lowering drugs (RR 1.8 (0.9–3.4)). Compared with the same reference group, the RR for breast cancer among women with past statin use was similar to that of current statin users, and past non-statin users had a risk that was nearly the same as that of current non-statin users.

Among current statin users, the median duration of treatment was 1.8 years and the maximum duration was 8.6 years. The data show no trend in risk with increasing duration of current statin use: the adjusted RRs of breast cancer were 1.1 (0.4–2.8) among six cases and 28 controls with current statin use of duration 3 to 5 years and 1.1 (0.4–3.0) among five cases and 26 controls with current statin use of duration longer than 5 years.

Current long-term use of hormone replacement therapy (HRT) was independently associated with a cumulative increase in risk (up to RR 2.1 (1.2–3.7) for women with 24 or more prescriptions compared with non-users; see Table 3), while past users had a somewhat decreased risk (RR 0.6 (0.4–1.0)). A history of benign breast disease was associated with an increased risk of breast cancer (RR 1.6 (0.8–3.1)). Women with BMI greater than 28 kg m^2^ had a slightly increased RR of 1.4 (0.9–2.1) compared to women with BMI less than 24 kg m^2^.

We considered that an association with hyperlipidaemia might occur spuriously if hyperlipidaemia were diagnosed incidentally during the evaluation of patients with suspicious breast lesions. Therefore, we restricted a secondary analysis to case–control sets in which the first diagnosis of hyperlipidaemia (if any) or prescription for a lipid-lowering drug (if any) was recorded 6 months or more before the index date. In this analysis of 1095 subjects (208 cases and 887 controls), the RRs among women with untreated hyperlipidaemia (1.9 (1.1–2.6)) and women who had hyperlipidaemia treated with non-statin drugs either currently (1.8 (0.9–3.9)) or in the past (2.0 (0.9–4.4)) were similar to those in the primary analysis. This additional analysis provided further evidence that there was no increased risk among women treated with statins either currently (RR 0.8 (0.5–1.4)) or in the past (RR 1.2 (0.6–2.6)).

## DISCUSSION

In this population-based, matched case–control study, neither current nor past use of HMG-CoA reductase inhibitors (‘statins’) showed any association with the risk of breast cancer. Longer duration of statin use was not associated with any increased risk of breast cancer.

Women with hyperlipidaemia who did not use lipid-lowering drugs or who used exclusively non-statin lipid-lowering drugs had a modestly increased risk compared to women without hyperlipidaemia. This is an unexpected finding. The few epidemiologic studies that have directly evaluated the relation of hyperlipidaemia to breast cancer risk have found little evidence for any association. In a follow-up study of over 95 000 women in the western US, no increased risk of breast cancer appeared following a single elevated serum cholesterol measurement at the time of enrolment (Hiatt *et al*, 1982) In a case–control study nested in the same cohort, there was no overall association between high-density lipoprotein cholesterol (HDL-C) and breast cancer, although modest indirect and direct associations were found for HDL-C among premenopausal and post-menopausal women, respectively (Moorman *et al*, 1998). The Framingham study found no association between cholesterol levels and the incidence of any cancer among women (Williams *et al*, 1981). However, cholesterol and triglyceride levels were increased among Greek women with breast cancer relative to controls (Alexopoulos *et al*, 1987).

The observed increase in risk of breast cancer related to hyperlipidaemia in our study was modest and could have arisen through unrecognized selection or information bias, unmeasured confounding, or by chance. It would therefore be inappropriate to conclude that statin treatment lowered the risk of breast cancer in our study, simply because women treated for hyperlipidaemia were at no greater risk than normolipemic women. There was no effect of the duration of current statin use on the risk of breast cancer, and the 95% confidence intervals for relative risk of breast cancer among women with untreated hyperlipidaemia and current statin users overlap considerably.

As reported in several other studies (Collaborative Group on Hormonal Factors in Breast Cancer, 1997; Colditz and Rosner, 2000; Schairer *et al*, 2000), current use of hormone replacement therapy (HRT) was associated with an increased risk of breast cancer, and the risk increased with the number of HRT prescriptions. We were not able to analyse reproductive risk factors because these data are not coded routinely in the GPRD. However, it is improbable that such factors would confound our results substantially because they are unlikely to determine anti-hypercholesterolemia treatment.
